# Systematic Evaluation of Clinical Efficacy and Platelet Function of Sofren Injection in the Treatment of Angina Pectoris

**DOI:** 10.1155/2021/5591137

**Published:** 2021-03-26

**Authors:** Genhao Fan, Menglin Liu, Zuoying Xing, Zhaoqi Chen, Mingjun Zhu, Yongxia Wang

**Affiliations:** ^1^Henan University of Chinese Medicine, Zhengzhou, Henan 450000, China; ^2^The First Affiliated Hospital of Henan University of Chinese Medicine, Zhengzhou, Henan 450000, China; ^3^Department of Traditional Chinese Medicine, Henan People's Hospital, Zhengzhou 450000, China

## Abstract

To systematically evaluate the efficacy and safety of sofren injection combined with conventional Western medicine in the treatment of angina pectoris. Randomized controlled trials (RCTs) on the treatment of angina pectoris with sofren injection combined with Western medicine were collected by searching PubMed, the Cochrane Library, Embase, Web of Science, CNKI, Wanfang Database, Weipu Database, and China Biomedical Literature Service System (CBM) by computer with the retrieval time from establishment of database to August 2020. After literature screening according to the predetermined inclusion and exclusion criteria, data of eligible studies were extracted, and then, a meta-analysis was conducted with the RevMan 5.3 software. The results of meta-analysis showed that the combination of sofren injection and Western medicine improved the platelet aggregation rate of patients (MD = −5.53, 95% CI (−6.42, −4.64), *P* < 0.00001), PAI-1 (SMD = −2.29, 95% CI (−2.57, −2.01), *P* < 0.00001), TXB2 (MD = −11.91, 95% CI (−14.50, −9.32), *P* < 0.00001), duration of angina attack (MD = −2.01, 95% CI (−3.14, −0.87), *P*=0.0005), ECG symptoms (RR = 1.29, 95% CI (1.20, 1.37), *P* < 0.00001), whole blood viscosity (MD = −1.07, 95% CI (−1.66, −0.48), *P*=0.0004), plasma viscosity (MD = −0.27, 95% CI (−0.35, −0.20), *P* < 0.00001), fibrinogen (MD = −0.67, 95% CI (−0.84, −0.50), *P* < 0.00001), whole blood high shear viscosity (MD = −1.04, 95% CI (−1.30, −0.79), *P* < 0.00001), whole blood low shear viscosity (MD = −2.03, 95% CI (−2.53, −1.53), *P* < 0.00001), CRP (MD = −1.96, 95% CI (−3.01, −0.91), *P*=0.0003), IL-6 (MD = −2.79, 95% CI (−4.02, −1.55), *P* < 0.00001), and TNF-*α* (MD = −17.34, 95% CI (−25.86, −8.81), *P* < 0.00001) and better than the Western medicine group, and there was no statistical significance in the incidence of adverse reactions between the two groups (*P*=0.48). The clinical application of sofren injection combined with conventional Western medicine in the treatment of angina pectoris is clear and safe, so it is recommended for clinical application.

## 1. Introduction

Ischemic heart disease is a leading cause of death and disability worldwide, and angina pectoris is its most common manifestation [1]. Angina pectoris is a clinical syndrome mainly characterized by paroxysmal chest pain or chest discomfort caused by insufficient blood supply of coronary artery, rapid temporary ischemia, and hypoxia of myocardium. According to the attack status and mechanism, it can be divided into stable angina pectoris (SA), unstable angina pectoris (UA), and variable angina pectoris (VA). It is estimated that the prevalence of angina in Western countries is 3-4% [2], and the prevalence increases with age in both men and women [3]. At present, the main therapeutic drugs include nitrate preparation, *β*-blockers, calcium channel blockers, coronary artery dilators, and antiplatelet drugs to improve the prognosis.

Sofren injection is a famous Chinese herbal medicine. It has the effect of promoting blood circulation, removing blood stasis, clearing arteries, and relieving pain. Its pharmacological effects include the anti-inflammatory effect and antiischemia effect, improving ECG changes, dilating coronary arteries, effectively reducing the load before and after the heart, improving cardiac function, reducing the degree of myocardial injury, reducing plasma viscosity, improving platelet function, and inhibiting thrombosis [4]. Conventional Western medicine treatment combined with sofren injection can increase the curative effect and improve the safety and the clinical symptoms. Most studies stopped in the period of clinical experience summary and the lack of a large sample of the prospective study, the pharmacological active ingredients, and pharmacological action mechanism still need further research. This study included the sofren injection for treatment of angina pectoris clinical RCTs. The effectiveness and safety of sofren injection in the treatment of angina pectoris was reviewed objectively by the systematic review method to provide evidence-based medical basis for its clinical application.

## 2. Materials and Methods

This meta-analysis followed the standard set of Preferred Reporting Items for Systematic Reviews and Meta-Analyses (PRISMA). The protocol for this study was registered with CRD42021234438.

### 2.1. Publication Retrieval Strategy

RCTs of sofren injection combined with conventional Western medicine in the treatment of angina pectoris were retrieved from PubMed, Embase, Cochrane Library, VIP, CNKI, Wanfang database, and China Biomedical databases since its establishment to August 2020. The subject word method was used for screening, and the Chinese retrieval words were sofren injection, coronary heart disease, and angina pectoris, while the English retrieval words were “Sofren injection,” “dazhuhongjingtian,” “*Rhodiola rosea*,” “Coronary heart disease (CHD),” and “Angina Pectoris.”

### 2.2. Type of Research

#### 2.2.1. RCTs of Sofren Injection in the Treatment of Angina Pectoris

*Research Objects*. According to “2012 ACCF/AHA Focused Update Incorporated Into the ACCF/AHA 2007 Guidelines for the Management of Patients with Unstable Angina/Non-ST-Elevation Myocardial Infarction” and “Guideline for the Diagnosis and Management of Patients with Stable Ischemic Heart Disease” [5, 6]. Consistent with the diagnosis of angina pectoris, age, gender, smoking, and alcohol history is not limited.

*Intervention*. The control group was only treated with the same conventional Western medicine (antiblood platelet, lipid regulation, anticoagulation, reducing the oxygen consumption of cardiac muscle, coronary expansion, and other basic treatment), while the treatment group was treated with sofren injection combined with conventional Western medicine.

*Observation Indexes*. Duration of angina symptoms, ECG improvement, platelet function (blood platelet aggregation rate, PAI-1, and TXB2), blood rheology (whole blood viscosity, plasma viscosity, fibrinogen, whole blood high shear viscosity, and whole blood low shear viscosity), serum factor (CRP, IL-6, and TNF-*α*), adverse drug reactions, or adverse events.

#### 2.2.2. Exclusion Criteria

The intervention measures did not meet the inclusion criteria, the diagnosis was not clear, the course of treatment was not clear, the test group only used sofren injection or the control group used other Chinese herbal medicines, and the outcome indicators did not include any of them except for adverse reactions.

#### 2.2.3. Literature Screening, Data Extraction, and Methodological Quality Assessment

Two independent researchers read full-text of the studies to extract relevant information, extract data content including literature, intervention methods, the basic situation of the bias risk assessment (type of study design, randomized methods, allocation concealment, blind method, the integrity of the data, and result report), relevant outcome indicators, and adverse reactions such as specific content; when the two researchers have a disagreement, it is discussed with a third party for evaluation, for the final documents for information extraction included in the literature on the basis of the Cochrane handbook [7] about clinical randomized controlled trial of bias in the risk assessment tools to evaluate, assess items with the stochastic method, and allocation concealment; participants and intervention provider were blinded implementation and outcome assessment results of blind method implementation, data integrity, selective, and other sources of bias; each of the above items were characterized according to the “low risk” (low), “high risk” (high), and “not clear” (unclear) for identification.

#### 2.2.4. Statistical Treatment

The included data were statistically analyzed using RevMan5.3 software. If the experimental results showed significant heterogeneity (*I*^2^≥50%), the random-effect model was used for meta-analysis. If the homogeneity of experimental results is good (*I*^2^<50%), and the fixed-effect model was used for meta-analysis. Risk ratio (RR) and 95% CI were used for the binomial variables. The mean difference (MD) and 95% CI were used when the continuous variables were the same unit of measurement. If measured by different methods or different units of measurement, it is expressed by the standardized mean difference (SMD). If a certain outcome indicators included in more than 10 references, it is through the funnel chart analysis whether there is a publication bias.

## 3. Results

### 3.1. Literature Search

**Figure 1:**
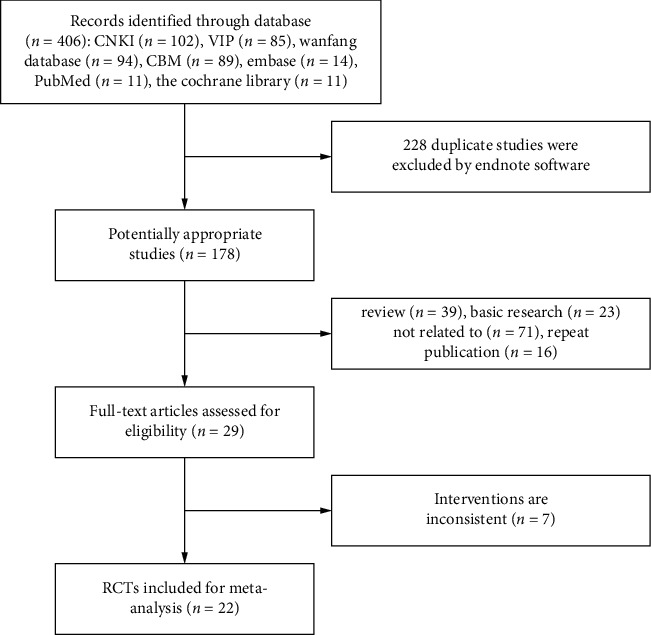
The document selection process and results.

A total of 406 articles were retrieved. The bibliographic titles retrieved from various databases were imported into EndNote X8, and a total of 22 included literatures were screened after repeated check by the software [8–29]. The literature screening process and results are shown in Figure 1.

### 3.2. Basic Characteristics of Literature Research

A total of 22 studies [8–29] were included, involving 2167 patients, including 1090 cases in the observation group and 1077 cases in the control group. No intergroup differences were found in all included studies. Among the indicators, 3 studies [8, 9, 11] observed the duration of angina attack, 11 studies [8, 10, 14–16, 18–20, 22, 23, 25] observed the improvement of ECG symptoms, 5 studies [12, 15, 27–29] observed platelet function, 6 studies [9, 10, 13, 19, 22, 24] observed hemorheology, 6 literatures [9–11, 15, 19, 20] observed serum factors, and 7 literatures [11, 13, 17–19, 21, 26] observed adverse drug reactions or adverse events, as shown in Table 1.

### 3.3. Quality Evaluation of Included Literature

**Figure 2:**
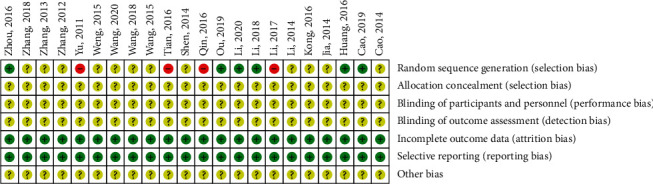
Bias risk assessment table.

The system evaluation was performed using the Cochrane handbook evaluation literature. The study included 22 articles, all into Chinese literature, including the 5 studies groups by the random number table method and 1 study using the lottery method; the risk of bias on the domain was judged as “low risk.” Three 4 studies had “high risk” because the random sequence was generated based on the date of visit or not described by the random method. The remaining 12 studies were reported in “random” without a specific method and were evaluated as “unclear risk;” all the research studies are not mentioned in the literature distribution hidden blind method. All data were complete and were included in the study of specific bias risk assessment information as shown in Figure 2.

### 3.4. Meta-Analysis Results

#### 3.4.1. Platelet Function

**Figure 3:**
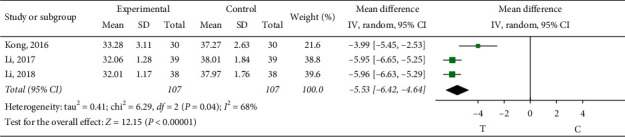
Meta-analysis of platelet aggregation rate in blood.

*Blood Platelet Aggregation Rate*. Three literatures [12, 15, 28] observed the platelet aggregation rate in blood. The heterogeneity test showed that there was significant heterogeneity among the results of studies (*P*=0.04, *I*^2^ = 68%), so the random-effects model was used for meta-analysis, and the results showed that the difference was a statistically significant (MD = -5.53, 95% CI (−6.42, −4.64), *P* < 0.00001), indicating that sofren injection had a better effect on improving the platelet aggregation rate of patients than the control group, as shown in Figure 3 In order to clarify the source of heterogeneity, literatures were excluded one by one through sensitivity analysis. When the study by Kong (2016) [28] was excluded (*p*=0.98, *I*^2^ = 0%), this study was the source of heterogeneity. Through reading the literature, it may be related to the patients themselves with other diseases.

**Figure 4:**
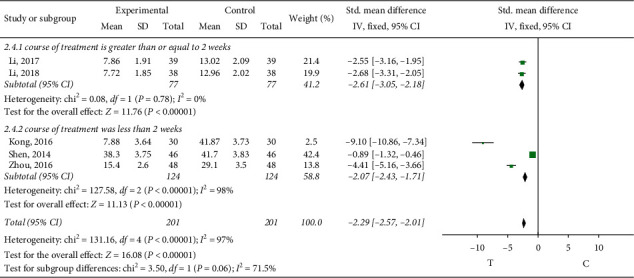
Plasma plasminogen activator inhibitor 1 meta-analysis.

*Plasma Plasminogen Activator Inhibitor 1 (PAI-1)*. Plasma plasminogen activator inhibitor 1 was observed in 5 studies [12, 15, 27–29]. Subgroup analysis was conducted according to different treatment courses, and the heterogeneity of treatment courses greater than or equal to two weeks was small, so the fixed-effect model was adopted for meta-analysis, and the results showed that the difference was statistically significant (MD = -2.61, 95% CI (−3.05, −2.18), *P* < 0.00001). The heterogeneity was greater when the treatment course was less than two weeks, the random-effects model was adopted for meta-analysis, and the results showed that the difference was statistically significant (MD = -2.07, 95% CI (−2.43, −1.71), *P* < 0.00001), indicating that sofren injection had a better effect on plasma plasminogen activator inhibitor 1 in patients than in the control group, as shown in Figure 4.

**Figure 5:**
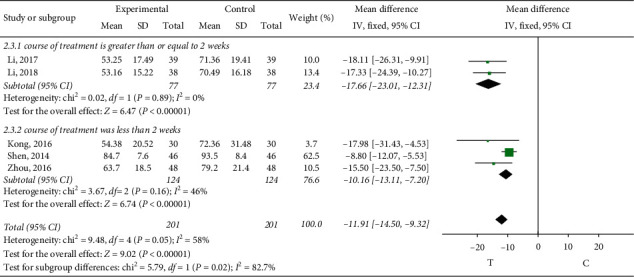
Meta-analysis of thromboxin B2.

*Thromboxin B2 (TXB2)*. Thromboxin B2 was observed in 5 studies [12, 15, 27–29]. Subgroup analysis was conducted according to different treatment courses, and the heterogeneity of treatment courses greater than or equal to two weeks was small, so the fixed-effect model was adopted for meta-analysis, and the results showed that the difference was statistically significant (MD = −17.66, 95% CI (−23.01, −12.31), *P* < 0.00001). The heterogeneity was small when the treatment course was less than two weeks, the fixed-effect model was adopted for meta-analysis, and the results showed that the difference was statistically significant (MD = −10.16, 95% CI (−13.11, −7.20), *P* < 0.00001), indicating that sofren injection had a better effect on the improvement of thromboxin B2 in patients than the control group, as shown in Figure 5.

#### 3.4.2. Duration of Angina Attack

**Figure 6:**
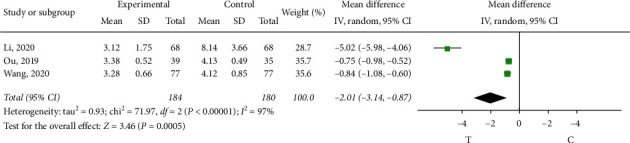
Meta-analysis of the duration of angina attack.

Three studies [8, 9, 11] observed the duration of angina attack. The heterogeneity test showed that there was significant heterogeneity among the results of studies (*P* < 0.00001, *I*^2^ = 97%), so the random effect model was adopted for meta-analysis. The results showed a statistically significant difference (MD = −2.01, 95% CI (−3.14, −0.87), *P*=0.0005) and showed that sofren injection in improving patients with angina duration of action is better than that of the control group, as shown in Figure 6. In order to clarify the source of heterogeneity, literatures were excluded one by one through sensitivity analysis. When the study by Li (2020) [9] was excluded (*p*=0.60, *I*^2^ = 0%), this study was indicated as the source of heterogeneity. Through reading the literature, it may be related to the patients themselves with other diseases.

#### 3.4.3. Improvement of ECG Symptoms

**Figure 7:**
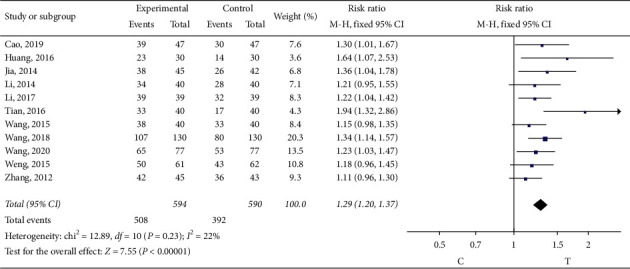
Meta-analysis of symptom improvement of electrocardiogram.

In 11 studies [8, 10, 14–16, 18–20, 22, 23, 25], the symptoms of electrocardiogram (ECG) were observed. The heterogeneity test (*P*=0.23, *I*^2^ = 22%) indicated small heterogeneity between the studies, so the fixed-effect model was adopted for meta-analysis. The results show that the difference is statistically significant (RR = 1.29, 95% CI (1.20, 1.37), *P* < 0.00001), suggesting that sofren injection in improving patients electrocardiogram of action is better than that of the control group, as shown in Figure 7.

#### 3.4.4. Hemorheology

**Figure 8:**
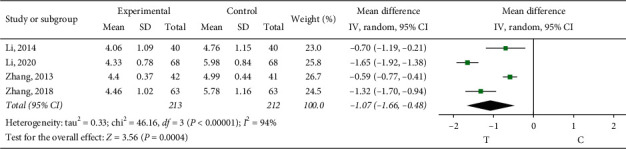
Meta-analysis of whole blood viscosity.

*Whole Blood Viscosity*. Four studies [9, 13, 22, 24] observed the whole blood viscosity. The heterogeneity test showed that there was significant heterogeneity among the results of studies (*P* < 0.00001, *I*^2^ = 94%); through reading the literature, the sources of heterogeneity may have been related to the conventional treatment regimen adopted in the study, so the random effect model was adopted for meta-analysis. The results showed a statistically significant difference (MD = −1.07, 95% CI (−1.66, −0.48), *P*=0.0004) and showed that the role of sofren injection in large strain improving patients' whole blood viscosity is better than that of the control group, as shown in Figure 8.

**Figure 9:**
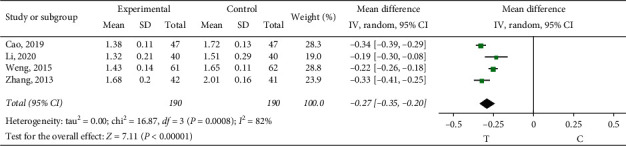
Meta-analysis of plasma viscosity.

*Plasma Viscosity*. Four studies [10, 19, 22, 24] observed the blood plasma viscosity. The heterogeneity test showed that there was significant heterogeneity among the results of studies (*P*=0.0008, *I*^2^ = 82%); through reading the literature, the sources of heterogeneity may have been related to the conventional treatment regimen adopted in the study, so the random effect model was adopted for meta-analysis. The results showed a statistically significant difference (MD = −0.27, 95% CI (−0.35, −0.20), *P* < 0.00001), suggesting that the role of sofren injection in improving patients' blood plasma viscosity is better than that of the control group, as shown in Figure 9.

**Figure 10:**
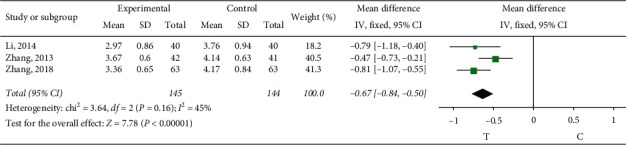
Meta-analysis of fibrinogen.

*Fibrinogen*. Three studies [13, 22, 24] observed the fibrinogen. The heterogeneity test (*P*=0.16, *I*^2^ = 45%) indicated small heterogeneity between the studies, so the fixed-effect model was adopted for meta-analysis. The results showed a statistically significant difference (MD = −0.67, 95% CI (−0.84, −0.50), *P* < 0.00001), suggesting that the role of sofren injection in improving the patients is better than that of the control group, as shown in Figure 10.

**Figure 11:**
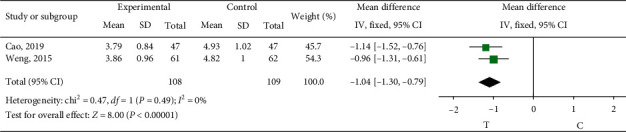
Meta-analysis of whole blood high shear viscosity.

*Whole Blood High Shear Viscosity*. Two studies [10, 19] observed the whole blood high shear viscosity. The heterogeneity test (*P*=0.49, *I*^2^ = 0%) indicated small heterogeneity between the studies, so the fixed-effects model was adopted for meta-analysis. The results showed a statistically significant difference (MD = −1.04, 95% CI (−1.30, −0.79), *P* < 0.00001), suggesting that the role of sofren injection in improving patients' whole blood high shear viscosity is better than that of the control group, as shown in Figure 11.

**Figure 12:**
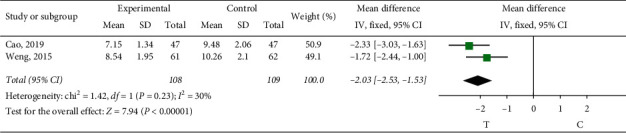
Meta-analysis of whole blood low shear viscosity.

*Whole Blood Low Shear Viscosity*. Two studies [10, 19] observed the whole blood low shear viscosity. The heterogeneity test (*P*=0.23, *I*^2^ = 30%) indicated small heterogeneity between the studies, so the fixed-effect model was adopted for meta-analysis. The results showed a statistically significant difference (MD = −2.03, 95% CI (−2.53, −1.53), *P* < 0.00001), suggesting that the role of sofren injection in improving patients' whole blood low shear viscosity is better than that of the control group, as shown in Figure 12.

#### 3.4.5. Serum Factors

**Figure 13:**
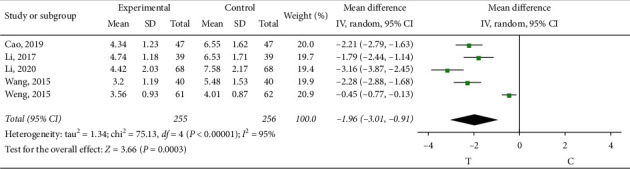
Meta-analysis of C-reactive protein.

*C-Reactive Protein (CRP)*. Five studies [9, 10, 15, 19, 20] observed CRP; the heterogeneity test showed that there was significant heterogeneity among the results of studies (*P* < 0.00001, *I*^2^ = 95%). The source of heterogeneity may be related to other chronic diseases associated with patients themselves, so the random effect model was adopted for meta-analysis. The results showed a statistically significant difference (MD = −1.96, 95% CI (−3.01, −0.91), *P*=0.0003) and showed that sofren injection in reducing patients' CRP is better than that of the control group, as shown in Figure 13.

**Figure 14:**
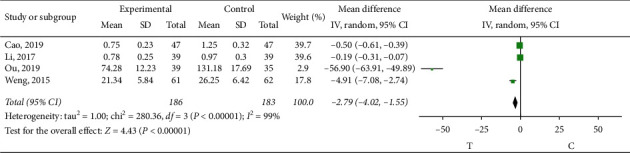
Meta-analysis of interleukin-6.

*Interleukin-6 (IL-6)*. Four studies [10, 11, 15, 19] observed IL-6; the heterogeneity test showed that there was significant heterogeneity among the results of studies (*P* < 0.00001, *I*^2^ = 99%). The source of heterogeneity may be related to the measurement method of the indicators, so the random effect model was adopted for meta-analysis. The results showed a statistically significant difference (MD = −2.79, 95% CI (−4.02, −1.55), *P* < 0.00001), suggesting that sofren injection in improving patients' IL-6 is better than that of the control group, as shown in Figure 14.

**Figure 15:**
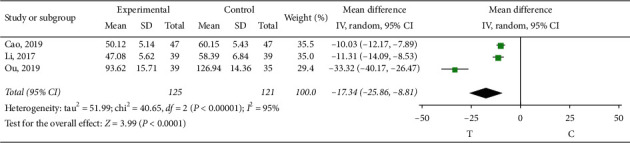
Meta-analysis of TNF-*α*.

*Tumor Necrosis Factor-αa(TNF-α)*. Three studies [10, 11, 15] observed TNF- *α*, and the heterogeneity test showed that there was significant heterogeneity among the results of studies (*P* < 0.00001, *I*^2^ = 95%). The source of heterogeneity may be related to the measurement method of the indicators. Therefore, the random-effect model was used for meta-analysis, and the results showed that the difference was statistically significant (MD = −17.34, 95% CI (−25.86, −8.81), *P* < 0.00001), indicating that sofren injection plays a better role in improving TNF-*α* than the control group (Figure 15).

#### 3.4.6. Adverse Reactions

**Figure 16:**
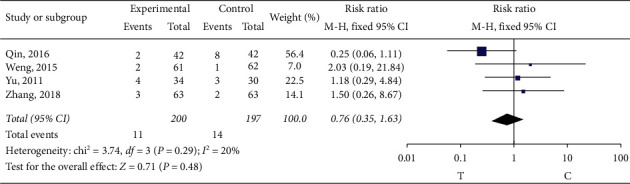
Meta-analysis of adverse reactions.

Seven studies [11, 13, 17–19, 21, 26] observed the adverse drug reactions or adverse events. Adverse reactions occurred in 4 of the studies [11, 15, 17, 24] (Table 2). The heterogeneity test (*P*=0.29, *I*^2^ = 20%) indicated small heterogeneity between the studies, so the fixed-effect model was adopted for meta-analysis. Meta-analysis showed no statistical significance (RR = 0.76, 95% CI (0.35, 1.63), *P*=0.48) and showed quite adverse reactions occurring between the two groups, as shown in Figure 16.

### 3.5. Risk Assessment of Bias

**Figure 17:**
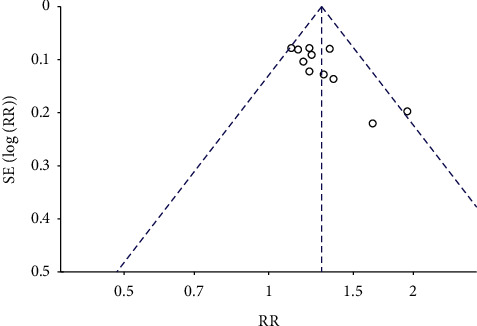
Infundibular diagram of therapeutic effect of electrocardiogram.

For the included literatures with more than 10 entries, the risk of publication bias was assessed for the outcome indicators. Funnel plots were observed, the ECG improvement outcome indicators showed incomplete symmetry on the left and right, suggesting the risk of publication bias, which may be related to the study quality and sample size of the included literatures, as shown in Figure 17.

## 4. Discussion

This systematic evaluation is mainly aimed at patients with angina pectoris treated by combining sofren injection on the basis of conventional Western medicine treatment to observe the duration of angina attack, improvement of ECG symptoms, platelet function, hemorheology, serum factors, adverse drug reactions, or adverse events. Results show that the sofren injection combined with conventional Western medicine improved the symptoms of patients with angina pectoris attack duration, electrocardiogram improvement, platelet function, blood rheology, and the serum factor effect and is better than that in the control group; there is no statistically significant difference in incidence of adverse reactions, but in the literature is less, and the result remains to be further validated.

Sofren injection contains rhodiola glucoside, tyrosol, polysaccharide, and other effective ingredients, which can dilate coronary artery and improve the ischemic tissue; therefore, sofren injection can relieve the symptoms of patients with angina pectoris and improve the ischemia status of ECG and at the same time can decrease the whole blood viscosity, inhibit thrombosis, and improve the function of platelet and clinical application of safety. Sofren injection is a kind effective in the treatment of coronary heart disease angina pectoris of Chinese herbal medicine; it can effectively make up for the inadequacy of the pure Western medicine treatment effect, reduce the dosage of Western medicine and the complications of patients. Sofren injection produces few side effects, and the forward curative effect is good, which can complement each other with Western medicine therapy and improve the survival rate and quality of life of patients. The adverse reactions of patients were also recorded in detail in this systematic evaluation to provide some evidence-based basis for clinical application in the future.

High-quality RCTs should be included in future studies because high-quality RCTs are a key factor in improving the level of evidence. During the implementation of the specific scheme, the random method and the estimation of sample size should be defined, the allocation concealment and blind method should be implemented, the lost follow-up cases should be recorded in detail, the adverse reactions of patients should be recorded in detail during the study period, and the research plan and scheme should be submitted for registration.

## 5. Conclusion

To sum up, sofren injection combined with conventional Western medicine can effectively improve the symptoms of patients duration of angina attack, electrocardiogram (ECG), platelet function, whole blood viscosity, plasma viscosity, fibrinogen, whole blood high shear viscosity, whole blood low shear viscosity, CRP, IL-6, and TNF-*α* and is superior to the pure Western medicine group. However, the overall quality of the included literature studies is low, so higher quality randomized controlled clinical trials are needed to further demonstrate the effectiveness and safety of sofren injection to provide better clinical guidance in the future.

## Figures and Tables

**Table 1:** Literature search.

Study IDs	Sample size		Duration	Intervention		Outcomes
	T	C		T	C	
Wang, 2020 [8]	77	77	2 w	Sofren injection 10 ml qd ivgtt + basic treatment	Basic treatment	①②
Li, 2020 [9]	68	68	40 d	Sofren injection 10 ml qd ivgtt + basic treatment	Basic treatment	①④⑤
Cao, 2019 [10]	47	47	2 w	Sofren injection 1 ml qd ivgtt + basic treatment	Basic treatment	②④⑤
Ou, 2019 [11]	39	35	2 w	Sofren injection 10 ml qd ivgtt + basic treatment	Basic treatment	①⑤⑥
Li, 2018 [12]	38	38	4 w	Sofren injection 10 ml qd ivgtt + basic treatment	Basic treatment	③
Zhang, 2018 [13]	63	63	2 w	Sofren injection 10 ml qd ivgtt + basic treatment	Basic treatment	④⑥
Wang, 2018 [14]	130	130	2 w	Sofren injection 10 ml qd ivgtt + basic treatment	Basic treatment	②
Li, 2017 [15]	39	39	2 w	Sofren injection 10 ml qd ivgtt + basic treatment	Basic treatment	②③⑤
Tian, 2016 [16]	40	40	2 w	Sofren injection 10 ml qd ivgtt + basic treatment	Basic treatment	②
Qin, 2016 [17]	42	42	10 w	Sofren injection 10 ml qd ivgtt + basic treatment	Basic treatment	⑥
Huang, 2016 [18]	30	30	2 w	Sofren injection 10 ml qd ivgtt + basic treatment	Basic treatment	②⑥
Weng, 2015 [19]	61	62	10 d	Sofren injection 10 ml qd ivgtt + basic treatment	Basic treatment	②④⑤⑥
Wang, 2015 [20]	40	40	2 w	Sofren injection 10 ml qd ivgtt + basic treatment	Basic treatment	②⑤
Cao, 2014 [21]	46	46	2 w	Sofren injection 10 ml qd ivgtt + basic treatment	Basic treatment	⑥
Li, 2014 [22]	40	40	15 d	Sofren injection 10 ml qd ivgtt + basic treatment	Basic treatment	②④
Jia, 2014 [23]	45	42	10 d	Sofren injection 10 ml qd ivgtt + basic treatment	Basic treatment	②
Zhang, 2013 [24]	42	41	10 d	Sofren injection 10 ml qd ivgtt + basic treatment	Basic treatment	④
Zhang, 2012 [25]	45	43	10 d	Sofren injection 10 ml qd ivgtt + basic treatment	Basic treatment	②
Yu, 2011 [26]	34	30	10 d	Sofren injection 10 ml qd ivgtt + basic treatment	Basic treatment	⑥
Zhou, 2016 [27]	35	35	10 d	Sofren injection 10 ml qd ivgtt + basic treatment	Basic treatment	③
Kong, 2016 [28]	58	54	4 d	Sofren injection 10 ml qd ivgtt + basic treatment	Basic treatment	③
Shen, 2014 [29]	26	24	8 d	Sofren injection 10 ml qd ivgtt + basic treatment	Basic treatment	③

①, duration of angina attack; ②, improvement of ECG symptoms; ③, platelet function; ④, hemorheology; ⑤, serum factor; ⑥, adverse drug reactions or adverse events.

**Table 2:** Adverse reactions.

Studies	Adverse drug reactions or adverse events	
	T	C
Zhang, 2018 [13]	1 case of shivering, 1 case of dizziness, and 1 case of nausea	1 case of dizziness and 1 case of nausea
Qin, 2016 [17]	1 case of dizziness and 1 case of nausea	2 cases of dizziness, 3 cases of headache, and 3 cases of nausea and vomiting
Yu, 2011 [26]	4 cases of headache	3 cases of headache
Weng, 2015 [19]	2 cases of mild dizziness and headache	1 case of mild dizziness and headache

## Data Availability

The datasets used and/or analyzed during the current study are available from the corresponding author upon request.
